# Self-Management Interventions for Kidney Transplant Recipients: A Systematic Review

**DOI:** 10.3390/healthcare13151918

**Published:** 2025-08-05

**Authors:** Hyejin Lee, Chan Mi Kang

**Affiliations:** 1 Department of Nursing, Research Institute of Dong-Eui Nursing Science, Dong-eui University, 176 Eomgwangno, Busanjin-gu, Busan 47340, Republic of Korea; hyejin.lee@deu.ac.kr; 2 Department of Nursing, Division of Health Science, Dongseo University, 47 jurye-ro, Sasang-gu, Busan 47011, Republic of Korea

**Keywords:** kidney transplantation, self-management, intervention, systematic review

## Abstract

**Background/Objectives**: For kidney transplantation, it is very important to provide effective post-transplantation interventions to help patients achieve continuous and efficient self-management. Therefore, we review the self-management interventions applied to kidney transplant recipients and suggest the optimal approach to increase the effectiveness of future self-management interventions. **Design**: Systematic review. **Methods**: Search terms and strategies included kidney transplantation; self-management; intervention; systematic review. We searched MEDLINE via PubMed, Excerpta Media dataBASE, Cochrane Register Controlled Trials, Cumulative Index to Nursing and Allied Health Literature, and one domestic Korean database to identify studies of self-management interventions for kidney transplant recipients aged ≥ 18 years published in English or Korean until 14 May 2025. Two reviewers independently selected related studies and extracted relevant data. Identified studies were assessed for quality and bias. **Results**: Of 1340 studies identified, 27 with 1912 participants met the inclusion criteria. Educational interventions were the most common self-management interventions and were provided 3 months to 1 year after kidney transplantation; most interventions were administered by nurses. Outcome variables were divided into cognitive, behavioral, affective, and health outcomes. Educational interventions were effective in improving cognitive, behavioral, and affective aspects. Some differences were observed, depending on the study. **Conclusions**: We recommend that nurse-involved educational interventions be included when developing self-management interventions and guidelines for kidney transplant recipients in clinical and community nursing settings.

## 1. Introduction

Kidney transplantation is considered the optimal treatment for patients with end-stage renal failure. In 2022, 25,498 people in the United States [1] and 2028 people in South Korea [2] underwent kidney transplantation, a procedure actively performed worldwide. However, kidney transplant does not completely cure the disease, even if the transplant is successful. Over time, infection, malignant tumors, and health problems (e.g., hypertension, diabetes, hyperlipidemia, muscle weakness, cardiovascular disease, and osteoporosis) may occur as a consequence of immunosuppressive therapy [3]. To prevent these complications and maintain function of the transplanted kidney, self-management strategies, such as accurate intake of immunosuppressants, early detection of organ rejection, prevention of complications, prevention of infection, dietary management, and daily life management, are essential [3,4]. Self-management is defined as actively participating in one’s own treatment process [5]. The treatment process for kidney transplant patients includes medication adherence, dietary management, infection control, and daily life management [6].

However, kidney transplant recipients often neglect disease management, fail to take their medications, or fail to carry out self-management interventions, including treatment instructions, as they find it difficult to deal with their disease [7,8]. In kidney transplant recipients, from early to late post-transplantation, non-compliance with immunosuppressants is 5.9–54.5% [9,10,11,12], the rate of non-adherence to dietary management is 34–85.3% [13,14,15], and the rate of non-adherence to daily life management (e.g., vital sign measurement and outpatient visits) is 16–95% [15,16,17,18]. In the United States, when immunosuppressant medications are not taken consistently, the medical cost per person increases by USD 12,840 for 3 years [11].

An immunosuppressant medication is essential for maintaining transplanted kidney function, but continuous use increases the risk of developing complications, such as infection, hypertension, and diabetes [19]. Hence, corresponding dietary management and daily life management should be implemented [20,21]. Effective self-management, such as adherence to the medication treatment, dietary management, and daily life management, can improve the health indicators within a short term and improve the quality of life by reducing the long-term risk of developing complications [20,21].

There are various methods of providing self-management interventions for kidney transplant recipients. With the recent development of information and communication technology, patient education at the time of discharge [22] and self-management interventions are implemented using mobile applications or artificial intelligence [23]. The interventions are provided as group or individual therapy. The outcome variables used for measuring the effectiveness of self-management interventions are reported in various categories including clinical outcomes and care process outcomes [23].

The interventions should be provided immediately after transplantation to enable patients to perform self-management interventions continuously and efficiently [21,24]. To develop effective interventions, preliminary studies should be conducted to analyze existing self-management interventions and methods of providing them. However, few literature reviews on the existing self-management interventions, such as information technology-based interventions and exercise training, have been conducted [23,25]. Moreover, studies that comprehensively analyze the composition or delivery method of all relevant interventions are limited.

Therefore, this systematic literature review aimed to examine the self-management interventions applied to early to late-stage kidney transplant recipients and to determine the strategies to increase their effectiveness in the future. Specifically, the types, components, and contents of self-management interventions applied to kidney transplant recipients were analyzed.

## 2. Materials and Methods

### 2.1. Study Design

This systematic literature review comprehensively analyzed the types, components, and contents of existing self-management interventions by integrating the self-management interventions applied to kidney transplant recipients. The review was conducted in accordance with the Preferred Reporting Items for Systematic Reviews and Meta-Analyses guidelines [26] (Tables S1 and S2). This review was pre-registered in the International Prospective Register of Systematic Reviews [PROSPERO] (registration date: 20 June 2022; registration number: CRD42022306551; Supplementary Materials S3). PROSPERO is an international database that can register research plans for systematic literature reviews; addresses issues, such as reporting bias and unnecessary repetition of research; and ensures transparency [27]. We aimed to reduce the potential bias in conducting and reporting research by pre-registering research protocols.

### 2.2. Search Methods

A literature search was conducted based on the following core question: ‘What self-management interventions are applicable to kidney transplant recipients and what are their types and component contents?’ All literature published until 14 May 2025 was searched. The search was performed according to the Core/Standard/Ideal model presented by the National Library of Medicine [28]. For core searches conducted overseas, MEDLINE via PubMed, Excerpta Media database (EMBASE), and Cochrane Central Register of Controlled Trials were used. For domestic searches, the Korean Studies Information Service System (KISS) and DataBase Periodical Information Academic were used. For overseas standard searches, the Cumulative Index to Nursing and Allied Health Literature (CINAHL) was used; for domestic searches, the Research Information Service System (RISS) was used. Additionally, the reference lists of all retrieved articles were reviewed to search for additional related studies.

The first preliminary search was conducted based on the type of study participants, interventions, comparisons, outcomes, and study design to select the appropriate search terms and establish search strategies appropriate for domestic and international data searches. Based on the Medical Subject Headings terms and text words used, AND/OR and truncation were appropriately applied. To search for studies conducted among target participants, the terms ‘kidney transplant*’, ‘renal transplant*’, ‘kidney replace’, ‘kidney graft’, ‘renal graft’, and ‘kidney transplantation’ were used during the search. The terms ‘self management’, ‘self care’, ‘self monitoring’, ‘self-administration’, ‘self guided’, ‘self help’, ‘self directed’, ‘self regulate’, and ‘self management’ were used to search for studies reporting the relevant interventions Supplementary Materials S4 shows the detailed search strategies.

Data retrieval, screening, and selection were independently conducted by two researchers with experience in numerous workshops related to systematic literature review and meta-analysis. In the event of disagreement, the two researchers reviewed whether the literature selection criteria were satisfied, and the items that did not match were selected after discussing their opinions with a third researcher. The third researcher was selected based on their experience with conducting multiple meta-analytical studies. The bibliographic information of all eligible studies was equally managed using RefWorks, and duplicates were excluded. After removing the duplicates, the titles and abstracts of the remaining studies were screened; studies that met the literature selection criteria were selected, while those that did not meet the selection criteria were excluded. The remaining studies that were not excluded were assessed for suitability by reviewing the original texts; the results of the assessment of excluded documents in each step were retained.

### 2.3. Inclusion and Exclusion Criteria

#### 2.3.1. Participants

This review evaluated studies that included kidney transplant recipients aged ≥18 years who were discharged after undergoing organ transplantation.

#### 2.3.2. Intervention

Studies that applied self-management interventions to kidney transplant recipients were selected. Self-management interventions refer to all interventions provided to kidney transplant recipients to promote self-management after undergoing transplantation. Studies selected included RCTs, quasi-experimental studies, and mixed methods studies.

#### 2.3.3. Comparisons

Since this review aimed to identify the self-management interventions applied to kidney transplant recipients, the presence or absence of a control group was not restricted.

#### 2.3.4. Outcomes

The results of this review were based on the quantitative values measured after providing self-management interventions to kidney transplant recipients. No restrictions were applied on the selection of search terms used as a result variable.

#### 2.3.5. Study Design

All types of studies that provided self-management interventions to kidney transplant recipients were included.

Studies that only presented protocols or abstracts and studies that were not published in Korean or English language were excluded.

### 2.4. Search Outcome

After searching the database using the appropriate search terms, 1303 non-Korean articles from MEDLINE via PubMed, EMBASE, the Cochrane Central Register of Controlled Trials, and CINAHL and 36 Korean articles from KISS, DBPia, and RISS were found. Moreover, the references were manually searched, and one relevant study was found; therefore, a total of 1340 articles were found during the search.

**Figure 1:**
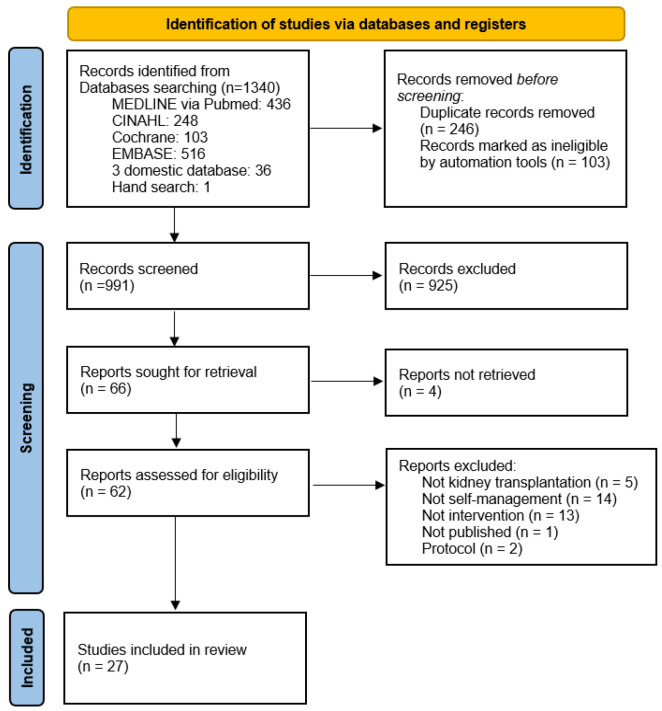
PRISMA flow diagram.

After removing the duplicates, 991 studies were retained. After the titles and abstracts were reviewed according to the inclusion and exclusion criteria, 66 studies were selected, and only 27 of these were included in the final review (Figure 1).

### 2.5. Quality Appraisal

The quality of studies included in the analysis was critically reviewed using the Joanna Briggs Institute (JBI; Adelaide, South Australia) quality assessment tool. The quality of randomized controlled studies was assessed using the JBI critical appraisal tool for randomized controlled trials [29], which comprised 13 items. The quality of quasi-experimental studies was assessed using the JBI critical appraisal tool for quasi-experimental studies [30], which comprised nine items. The questions were rated as ‘yes’, ‘no’, ‘unclear’, and ‘not applicable’. A ‘yes’ response indicated that the JBI quality evaluation criteria were met. The JBI quality evaluation tool does not contain the quantitative criteria for evaluating the quality of studies; instead, it examines the appropriateness of the evaluation items. Therefore, after the two independent researchers had evaluated each other’s assessment findings, quality evaluation was conducted via review and discussion until a consensus was reached. Twenty studies were selected and rated as follows: 1 point for a ‘yes’ response and 0 points for a ‘no’ or an ‘unclear’ response. When the number of ‘yes’ responses was more than half of the total score, the studies were selected for systematic review [31].

### 2.6. Data Abstraction

To analyze the characteristics of self-management interventions, data on the intervention type, author’s name, year of publication, providers, targets, duration of the interventions, and content of the intervention were extracted and used as a basis for analyzing the selected studies.

### 2.7. Synthesis

Two researchers, including the author, independently analyzed the characteristics of all selected studies and presented them in a coding table. The coding table contained the author’s name, year of publication, country of publication, study design, number of participants, average age of participants, type of intervention, intervention of control group, and outcome variable. If the two reviewers provided different opinions on the selection and exclusion criteria, the original text was reviewed by a third expert, and the disagreement was resolved by consensus.

## 3. Results

### 3.1. General Characteristics of the Selected Literature

Table 1 shows the general characteristics of the 27 selected studies. Five studies were conducted in Iran (18.5%), five in the United States (18.5%), four in China (14.8%), three in South Korea (11.1%), one in Germany (3.7%), one in the Netherlands (3.7%), one in France (3.7%), one in Italy (3.7%), one in Switzerland (3.7%), one in Poland (3.7%), one in Pakistan (3.7%), one in Kuwait (3.7%), one in Thailand (3.7%), and one in Canada (3.7%). The publication years ranged from 2012 to 2024, with four studies (14.8%) conducted in 2018, 2022, and 2023. Analysis of the study design identified 16 randomized controlled trials (RCTs) (59.3%), nine quasi-equivalent studies (33.3%), and one mixed method study and retrospective study (3.7%), respectively. The review included 1912 participants, of whom 1083 were assigned to the experimental groups and 829 assigned to the control groups. For the control groups, 11 studies (40.7%) involved the provision of general nursing care, while two studies (7.4%) did not involve the performance of any intervention. The outcome variables for self-management among kidney transplant recipients included quality of life, self-management knowledge and behavior, self-care, self-efficacy, treatment adherence, satisfaction level, burden, anxiety, depression, uncertainty, biomedical parameters (HbA1c, lipid profile, blood pressure, renal function tests, body mass index, etc.).

### 3.2. Methodological Quality Assessment of Literature

Table 2 shows the quality evaluation results of 27 studies. All 16 RCTs were randomized. Thirteen studies included groups with similar characteristics at baseline [20,32,33,39,43,44,45,46,50,51,53,54,55], six studies involved blinding of the participants to the interventions [32,36,45,48,49,53], seven studies involved blinding of the treatment providers [33,45,48,49,50,51,53], and seven studies involved blinding of the assessors [32,33,48,49,50,51,55]. Nine studies explained the reasons for dropouts and the differences between the groups due to dropouts [33,36,39,43,44,45,48,49,53]; meanwhile, 14 studies conducted an intention-to-treat analysis [20,32,33,36,39,43,44,46,48,49,50,51,53,54]. Reliable measurements and analyses were conducted in all studies. Of the ten quasi-experimental studies [21,35,37,38,40,41,42,47,52,56], the cause and effect were clearly presented in all studies, and four studies had control groups [36,38,48,56]. Both groups showed similar characteristics at baseline. The results of all ten studies were accurately measured in all study groups using a reliable method, and statistical analyses were appropriately performed. As JBI does not provide a mixed-method research checklist, and this review focused on examining the interventions, the JBI quasi-experimental research checklist was applied in one study [41]. The participants responded ‘yes’ to seven out of the nine items in the study. None of the studies were excluded based on methodological quality.

### 3.3. Self-Management Interventions for Kidney Transplant Recipients

Table 3 shows the characteristics of self-management interventions applied to kidney transplant recipients. Self-management educational interventions were the most common (44.4%), followed by exercise interventions (11.1%) and education combined with counseling interventions, and mobile apps (7.4% each). Education combined with an exercise intervention, mobile app combined with nursing coaching, counselling, medication monitoring, motivational enhancement, empowerment enhancement, self-management support, and behavioral educational interview interventions were also provided (3.7% each).

The providers of interventions included nurses in ten studies (37.0%); nurses, psychologists, and dietitians, or nurses, public health workers, and pharmacists, or nurses, dietitians, and pharmacists or physicians and clinical psychologists in one study (3.7%); nurse–doctor or nutritionist or mobile app and nurse in two studies (7.4%); physicians in one study (3.7%); pharmacists in one study (3.7%); mobile app in one study and web, mobile phones, and tablets in five studies (18.5%).

The participants who received interventions included patients treated immediately after undergoing kidney transplantation [21,38,41,53,54] and patients treated within 20 years after undergoing kidney transplantation. Five studies included individuals who were treated within 3 months after undergoing kidney transplantation [20,33,43,44,46], while three studies included individuals who were treated within 1 year after undergoing kidney transplantation [34,35,45]. One study included individuals who were treated within 6 months after undergoing kidney transplantation [48]. The duration of the intervention ranged from the time the patient waited for an outpatient doctor [50] until 12 months [42,51], with 3 months being the most common intervention period reported in nine studies [20,33,39,44,45,47,48,49,55].

The contents of educational interventions included information on the method of administration, therapeutic and side effects of the medications, diet, physical and self-care activities, problem solving, emotional control, social interaction [20,21,32,33,35,38,40,43,48,53]; the methods of maintaining effective relationships with family, friends, and healthcare professionals [43]; nutrition management [21,32,41,42,43,51,52,53]; exercises and activities [32,41,42,51,52,53]; evaluation of new therapies [43]; blood concentration monitoring [20,52]; prevention and treatment of complications [20,41,42,52,53]; rejection management [41,42]; self-care activities [21,45]; methods of detecting unusual symptoms after undergoing kidney transplant and the corresponding management [45]; stress management [38,45,53]; skin cancer prevention strategies [50]; emotional or mental health management [51]; weight management [51]; and healthcare in daily life [42,52]. The exercise interventions included aerobic and strength training exercises and dietary management [37], provision of assistance and improvement in physical activities [46], maintenance or achievement of normal body weight, and the integration of physical activity into the individual’s daily routine [51]. In particular, web-, mobile phone-, or tablet-based interventions included those that enhance individual motivation [34]; health knowledge related to each stage of renal transplantation [56]; self-monitoring [56]; nurse–patient communication [56]; monitoring diet and physical activity [47]; help cope with the medication side effects; control events that may interfere with the effects of medications; promote good interaction between patients and healthcare providers [36]; involve the provision of medication reminders, making phone calls, and sending text messages [44]; and allow the provision of skin cancer prevention education using the applications installed in tablets [50].

### 3.4. Outcomes of Self-Management Interventions Applied to Kidney Transplant Recipients

The outcome variables that provided self-management interventions were divided into four categories (Table 4): cognitive outcomes (e.g., treatment knowledge, and self-management knowledge), behavioral outcomes (e.g., self-management behavior, medication adherence, self-management behavior, and daily step), affective outcomes (e.g., quality of life, feelings after kidney transplantation, adherence to immunosuppressive medications, satisfaction, and anxiety), and health outcomes (e.g., blood pressure, heart rate, and body mass index).

The four effective cognitive outcomes of self-management interventions were treatment knowledge [35], self-management knowledge [21,48,52], knowledge of skin cancer and sun protection [50], and perception of care [51]. Conversely, the self-management interventions did not affect the self-perceived general health status [36]. The effects of self-management interventions on quality of life varied between studies.

The effective behavioral outcomes of self-management interventions included enhanced self-care ability [20,54], sun protection behavior [50], and treatment compliance [42]; however, they did not affect medication intake-related skills [36] and physical activity [51,55]. The effects of self-care behavior, medication adherence, and daily steps varied between studies.

Self-management interventions were effective in improving the affective outcomes including feelings after kidney transplantation, adherence to immunosuppressive therapy [34], perceived level of empowerment [39], satisfaction level [20], self-perceived burden [20], stress [47], and level of uncertainty [42]. However, these interventions did not affect the patients’ comfort levels [54].

The effective health outcomes included heart rate [46], HbA1c [48], and waist circumference [46], but they did not affect the results of the six-minute walking test [46], medication side effects [36], or myocardial function [37]. The effects on blood pressure, body mass index, and renal function varied between studies.

## 4. Discussion

This review aimed to systematically examine the self-management interventions provided to kidney transplant recipients and determine strategies that can help increase their effectiveness. In this section, we focus our discussion on the key findings identified through the review.

About 44% of the included studies were conducted in Iran, China, and Korea. All three countries have a high prevalence of end-stage renal disease and are countries where kidney transplantation is widely performed. As the importance of self-management is emphasized for long-term survival and quality of life after transplantation, self-management intervention studies are actively being conducted in Iran, China, and Korea [57,58,59]. In addition, these countries have high national interest in kidney disease management and transplant patient management, and self-management-related studies are actively being conducted centered on university hospitals and research institutes [6]. Additional analyses considering sociocultural factors in each country will be necessary.

### 4.1. Types of Self-Management Interventions for Kidney Transplant Patients: Educational Interventions and Mobile Apps

With regard to the type of self-management interventions applied to kidney transplant recipients, various interventions (e.g., educational intervention, exercise intervention, and counseling intervention) were applied, the majority of which were education-related. This finding supports the result of previous studies, which indicated that kidney transplant recipients had high educational needs owing to the complicated post-kidney transplant management [60]. After kidney transplantation, patients require knowledge about medication, rejection management, complication management, and infection prevention to maintain the function of the transplanted kidney [61,62,63]. Insufficient patient knowledge can lead to serious consequences, such as rejection of transplanted organs due to low adherence with immunosuppressant therapy and therapeutic management [63]. Sufficient knowledge regarding kidney transplantation has been shown to reduce morbidity and mortality rates, as well as the costs associated with healthcare and disease management [64]. Accordingly, education is a critical component of self-management interventions, as it enhances patient understanding and engagement. A previous meta-analysis of interventions for hypertensive patients reported that 66.7% of studies included educational components, and those combining education, counseling, and management strategies were particularly effective in lowering blood pressure [65].

In developing future self-management interventions for kidney transplant recipients, education should serve as a key modality for knowledge transfer. Core educational content typically includes medication adherence, nutrition, physical activity, complication monitoring, and stress and emotional management. These elements must be effectively communicated, and mobile technologies offer a highly accessible and scalable platform for delivery. However, beyond mere knowledge dissemination, it is crucial to ensure patient adherence to self-management behaviors. Therefore, incorporating nurse-led monitoring and coaching alongside mobile interventions may yield more effective outcomes than relying on mobile applications alone.

### 4.2. Self-Management Intervention Methods for Kidney Transplant Patients

Nurses were the most common intervention providers. In a preliminary review study [66] analyzing how nurse-led interventions can help improve self-management skills among outpatients with chronic conditions, nurses were identified as the most effective providers of self-management interventions in specific settings and for specific patient populations. Since nurses are at the forefront of patient management, they can quickly identify symptoms, discomfort, and difficulties in performing self-management and promote active self-management. The role of nurses is especially important within the first year after organ transplantation until the patient is able to perform self-management independently. The duration of intervention ranged from 3 months to 1 year after kidney transplantation. This is intended to help patients quickly learn unfamiliar and complex life activities after undergoing kidney transplantation and apply them in their daily lives. Educational intervention was provided multiple times rather than one time. This finding suggests that repeated education is important not only because of the need to share different contents with the target patients but also because of the need to establish different behavioral guidelines as part of daily life (habit formation) after undergoing kidney transplantation.

### 4.3. Self-Management Intervention Effects for Kidney Transplant Patients

The effectiveness of actual self-management interventions for kidney transplant recipients was evaluated using various outcome variables depending on the type of intervention used. In the cognitive outcome domain, the most frequently reported significant effects of self-management interventions were self-management knowledge [21,48,52] and quality of life [20,32,33,40,43,54,56]. Repetitive education improved knowledge of lifestyle factors such as diet and exercise [48], and individual education programs enhanced knowledge of medication management, dietary management, weight management, complication management, physical activity, and stress management [52]. Additionally, the multidisciplinary self-management education program improved self-management knowledge in medication management, dietary management, exercise, and infection prevention [21]. These repetitive individual or multidisciplinary education programs help improve knowledge, which is important for their health management. The self-management program improved both physical health and emotional well-being, leading to enhanced overall quality of life as patients improved their self-management skills [43]. The quality of life was positively affected by rapid surgical recovery nursing and continuous nursing care [54], and the quality of life was improved in both mental health (reduction in depression and stress levels) and physical health (weight, blood sugar management, etc.) as weight loss was achieved through cognitive–behavioral therapy and dietary control weight loss intervention [33]. Although one study found no effect on quality of life [34], indicating the need for repeated studies, most research results suggest that improvements in quality of life have a significant impact on patients’ long-term treatment adherence and health.

In the behavioral outcome domain, the most frequently reported significant effects of self-management interventions were self-care behavior [39,40,45,56] and self-care ability [20,54]. Specifically, the interventions led to an increase in self-efficacy [20,39,54], which significantly improved self-management behaviors such as medication adherence [39,40,45,56], diet management [39,40,45,56], weight monitoring [39], blood pressure monitoring [39,56], blood sugar monitoring [40,56], and exercise [40,56]. Mobile health application interventions were important in continuously adjusting and improving behaviors by providing real-time feedback [56]. Nurse-led self-management support was also reported to play a crucial role in positively changing self-management behaviors [40]. Nursing interventions based on the Health Belief Model and continuous nursing were effective in enhancing patients’ self-management abilities (medication adherence and diet management) [20,54].

In the health outcome domain, the most frequently reported significant effects of self-management interventions were waist circumference (WC) [46,48] and weight [33,47]. Repetitive and structured education [48] and cognitive–behavioral therapy [33] influenced the lifestyle knowledge and self-management of kidney transplant patients, resulting in effective reductions in waist circumference and weight. Cognitive–behavioral therapy helps improve self-control and manage stress, which in turn helps prevent unnecessary overeating. However, there are results showing that BMI, related to WC and weight, was not effective [51,55], indicating the need for further research.

The effect of the intervention type on the outcome variable was confirmed. Educational intervention was effective in improving the cognitive, behavioral, and affective outcomes, while exercise intervention was effective in improving the cognitive and health outcomes. Changes in behavioral results were expected after providing the exercise intervention, but this type of intervention did not affect the outcome variables; hence, further studies are warranted to evaluate the effectiveness of exercise interventions.

Since self-management interventions after kidney transplantation are diverse and complex, a variety of tailored approaches are necessary to help recipients maintain a healthy life after transplantation [60]. Accordingly, to actively utilize evidence-based interventions for which the effects have been verified, including self-management education, multidisciplinary considerations and cooperation are required in the clinical and community nursing settings [21]. In particular, in studies conducted in the last two years, the tendency of various occupations such as nurses, pharmacists, dietitians, and clinical psychologists to provide interventions was remarkable [21]. Various aspects of self-management after kidney transplantation should be considered, and multidisciplinary management is essential for this.

To properly interpret and apply the results of this review, the following limitations should be considered. First, since this review did not statistically analyze the effect sizes, it is difficult to objectively compare the effects according to the outcome variables. Hence, a meta-analysis of the effect size should be conducted in the future. Second, although this review presents various self-management interventions, measurement variables, and tools, the number of relevant studies is relatively small to make a comparison of the effects of each intervention; hence, caution should be observed when interpreting the results, and further studies are needed. Third, this study included only studies published in English and Korean, so future studies including multiple languages are needed. Fourth, since this systematic literature review was only conducted in published studies, a publication bias may exist owing to the exclusion of unpublished studies. Fifth, we conducted a systematic literature review to identify the overall content of self-management interventions provided to kidney transplant patients. Results showed that the outcome variables and measurement tools used in each study were diverse, and the number of studies was small, so a formal analysis of heterogeneity was not performed. As the number of studies increases in the future, we recommend that heterogeneity by study design, participant, intervention type, and follow-up period be identified and meta-analysis performed accordingly.

This review has some strengths. First, this review attempted to determine the overall status and contents of interventions by comprehensively identifying the types of self-management interventions applied to kidney transplant recipients. Since efforts were made to provide information on the factors considered and the outcome variables measured when constructing future interventions, sufficient baseline data were used to develop effective interventions. Second, the quality assessment was performed to increase the accuracy of the results of the included studies, and most of the 27 studies were randomized controlled trials and quasi-equivalent experimental studies, showing high quality, so the risk of bias was low, and the strength of the evidence was high. This increases the reliability of the results of this review, which analyzed the effects of self-management interventions for kidney transplant recipients and can serve as a reliable basis for selecting the appropriate interventions for kidney transplant recipients in the future.

## 5. Conclusions

This review aimed to identify the main contents, methods, and effects of self-management interventions applied to kidney transplant recipients through a systematic literature review. The literature analysis revealed that studies using educational intervention are actively progressing, and a complex application of various interventions might be required in the future. Specifically, most intervention providers are nurses, who can quickly identify and respond to symptoms reported by patients or difficulties in self-management during patient management; therefore, they promote active self-management among patients. Self-management interventions for kidney transplant recipients had a positive effect on patients’ cognitive, behavioral, affective, and health outcomes. In particular, educational intervention was effective in improving the patients’ cognitive, behavioral, and affective outcomes. The results of this review encourage the inclusion of educational interventions involving nurses in the development of self-management interventions and guidelines for kidney transplant recipients in clinical and community nursing settings.

## Figures and Tables

**Table 1:** Descriptive summary of all included studies (n = 27).

Study First Author (Year, Country)	Research Design	Patient Sample Size (n)	Patients’ Age (Mean /Median)			Intervention	Control	Outcomes
		I	C	I	C			
Aghakhani et al. (2021, Iran) [32]	RCT	29	30	42.7	41.5	Self-care education program	Routine care	(1) Quality of life
Barchfeld et al. (2023, Germany) [33]	RCT	28	28	48.2	47.7	Cognitive–behavioral intervention (nutritional counseling)	Brief self-guided intervention	(1) Percentage of weight loss (2) BMI (3) Renal function parameters (4) Quality of life (5) Levels of depression and anxiety
Been-Dahmen et al. (2019, Netherlands) [34]	Mixed-method design	24	33	59.7	59.8	Nurse-led self-management intervention	Routine care	(1) Self-management knowledge and behavior (2) Quality of life (3) Self-efficacy (4) Feelings after kidney transplantation (5) Quality of nurse-led care (6) Social support (7) NPs’ fidelity to intervention protocol (8) Importance vs. actual attention to topic during nurse-led consultation session
Chambord et al. (2021, France) [35]	Before and after comparative study	44	48	59.5	55.0	Pharmacist-led interventions	Routine care	(1) Treatment knowledge (2) Medication adherence (3) Measurement of drug exposure
Côté et al. (2018, Canada) [36]	RCT	23	16	54.0	51.4	Web-based tailored education	Conventional transplantation-related websites	(1) Medication adherence (2) Self-efficacy (3) Medication intake-related skills (4) Medication side effects (5) Self-perceived general state of health
Enrico et al. (2018, Italy) [37]	Before and after comparative study	30	-	38.6	-	Supervised exercise program	N/A	(1) Myocardial function (2) Functional assessment
Hoseinian et al. (2023, Iran) [38]	Quasi-experimental study	30	30	63.6	63.4	Education based on the model of individual health promotion strategies	Routine care	(1) Self-care self-efficacy - Stress reduction - Adaptation - Decision making - Enjoying life
Hsiao et al. (2016, China) [39]	RCT	56	66	48.6	45.9	Empowerment support program	Routine care	(1) Perceived level of empowerment (2) Self-care behavior
Hu et al. (2022, China) [20]	RCT	30	30	45.96	45.91	Nursing intervention based on health belief model	Routine care	(1) Satisfaction (2) Self-perceived burden (2) Drug compliance (3) Anxiety (4) Depression (5) Self-management ability (6) Quality of life
Jabeen et al. (2021, Pakistan) [40]	Quasi experimental study (Before and after)	36	-	31.4	-	Nurse-led self-management program	N/A	(1) Quality of life
Jeong et al. (2021, South Korea) [41]	Before and after comparative study	30	-	49.1	-	Theory-based self-management program	N/A	(1) Autonomy support (2) Competence (3) Self-care agency
Kim et al. (2017, South Korea) [42]	Quasi-experimental study (Nonequivalent control group pre-posttest design)	25	28	45.48	46.43	Empowerment education program	None	(1) Uncertainty (2) Self-care agency (3) Compliance
Kuwaiti et al. (2018, Iran) [43]	RCT	33	34	46.3	43.2	Chronic disease self-management training program	Attending one training session on diet	(1) Quality of life
McGillicuddy et al. (2013, USA) [44]	RCT	9	10	42.4	57.6	Mobile phone-based medication monitoring	Education related to post- transplantation medical care	(1) Medication adherence
Mollazadeh et al. (2018, Iran) [45]	RCT	42	37	41.27	38.0	Teach-back training-based education	Unknown	(1) Self-management
O’Brien et al. (2020, USA) [46]	RCT	25	25	65.7	65.1	SystemCHANGE + activity tracker intervention	Received transplantation-related educational materials	(1) Daily steps (2) Health outcomes - Blood pressure - Heart rate - Body mass index - Waist circumference - Physical function (6 min walk test)
O’Brien et al. (2024, USA) [47]	Before and after comparative study	20	-	59.5	-	Technology, Application, Self-Management for Kidney (TASK) intervention	N/A	(1) Blood pressure (2) Weight (3) Fruits/vegetable intake, fiber intake, sodium intake (4) Self-efficacy to exercise (5) Perceived stress
Othman et al. (2024, Kuwait) [48]	RCT	140	70	44.9	44.0	Structured diabetes education	Conventional education	(1) Knowledge - Healthy food knowledge - Exercise knowledge - Healthy foot care (2) Biochemical parameters - HbA1c - Lipid profile - Renal function tests - Fasting sugar - Weight, BMI, and waist circumference (3) Diabetes self-care
Pollock et al. (2023, USA) [49]	RCT	7	9	41.6	35.9	Self-management app (MyKidneyCoach), tailored text, telephonic clinical nurse coaching	Self-management app (MyKidneyCoach)	(1) Patient activation measure (2) Partners in health (self-management) (3) Nutrition self-efficacy score
Robinson et al. (2015, USA) [50]	RCT	84	86	51.0	49.0	Educational sun protection program	Received general skin care information	(1) Knowledge of skin cancer and sun protection (2) Attitudes - Recognition of personal risk to skin cancer - Willingness to change sun protection behavior (3) Sun protection behavior - Sun protection - Daily number of hours staying outdoors
Schmid-Mohler et al. (2019, Switzerland) [51]	RCT	61	62	50.5	49.8	Educational Weight Management Intervention	Routine care with brochure	(1) BMI (2) Body composition (LTM) (3) WHR (4) Physical activity - Self-reported physical activity - Number of steps (5) Perception of care
Sim et al. (2012, South Korea) [52]	Quasi-experimental study (Nonequivalent one group pre-posttest design)	42	-	47.4	-	Individual educational program	N/A	(1) Self-care knowledge (2) Self-care behavior
Soltannezhad et al. (2013, Iran) [53]	RCT	26	26	38.12	37.65	Educating health promotion strategies	Routine care	(1) Self-care self-efficacy
Song et al. (2022, China) [54]	RCT	30	30	31.85	32.59	FTS nursing combined with continuous nursing	FTS nursing	(1) Patients’ comfort (2) Self-care ability (3) Medication compliance (4) Quality of life
Thangto et al. (2022, Thailand) [21]	Before and after comparative study	50	-	39.0	-	Multidisciplinary education program	N/A	(1) Knowledge - Self-care knowledge - Nutrition and dietary knowledge - Immunosuppressive drugs knowledge
Wesołowska-Górniak et al. (2022, Poland) [55]	RCT	49 †	51 †	33.8	36.2	Self-monitoring of daily physical activity using a pedometer	None	(1) Average daily number of steps within 7 days (2) Physical activity (3) Body composition - BMI - Fat% - FFM
Xie et al. (2023, China) [56]	Retrospective study	80	80	-	-	Mobile medical application self-management behavior intervention	Conventional self-management behavior intervention	(1) Self-management behaviors (2) Quality of life (3) Self-efficacy

BMI = body mass index; Fat% = percentage of body fat; FFM = free fat mass; FTS = fast-track surgery; KTx = patients after kidney transplantation; LTM = lean tissue mass; N/A = not applicable; RCT = randomized clinical trial; WHR = waist–hip ratio; † Liver transplantation recipients were included.

**Table 2:** JBI critical appraisal of all included studies by research design.

Design/Citation	Critical Appraisal													
	Q1	Q2	Q3	Q4	Q5	Q6	Q7	Q8	Q9	Q10	Q11	Q12	Q13	Total
**RCTs**														
Aghakhani et al. (2021) [32]	Y	Y	Y	Y	N	Y	Y	N	Y	Y	Y	Y	N	10/13 ^†^
Barchfeld et al. (2023) [33]	Y	UC	Y	N	N	UC	Y	Y	Y	Y	Y	Y	Y	9/13 ^†^
Côté et al. (2018) [36]	Y	Y	UC	Y	Y	Y	Y	Y	Y	Y	Y	Y	Y	12/13 ^†^
Hsiao et al. (2016) [39]	Y	Y	Y	UC	UC	UC	Y	Y	Y	Y	Y	Y	UC	9/13 ^†^
Hu et al. (2022) [20]	Y	UC	Y	UC	UC	UC	Y	UC	Y	Y	Y	Y	UC	7/13 ^†^
Kuwaiti et al. (2018) [43]	Y	UC	Y	UC	UC	UC	Y	Y	Y	Y	Y	Y	UC	8/13 ^†^
McGillicuddy et al. (2013) [44]	Y	UC	Y	UC	UC	UC	Y	Y	Y	Y	Y	Y	N	8/13 ^†^
Mollazadeh et al. (2018) [45]	Y	Y	Y	Y	Y	UC	Y	UC	UC	Y	Y	Y	Y	10/13 ^†^
O’Brien et al. (2020) [46]	Y	Y	Y	UC	UC	UC	Y	Y	Y	Y	Y	Y	Y	10/13 ^†^
Othman et al. (2024) [48]	N	N	UC	Y	Y	Y	Y	Y	Y	Y	Y	Y	Y	10/13 ^†^
Pollock et al. (2023) [49]	N	N	N	Y	Y	Y	Y	Y	Y	Y	Y	Y	Y	10/13 ^†^
Robinson et al. (2015) [50]	Y	Y	Y	N	Y	Y	Y	N	Y	Y	Y	Y	N	10/13 ^†^
Schmid-Mohler et al. (2019) [51]	Y	Y	Y	N	Y	Y	Y	UC	Y	Y	Y	Y	UC	10/13 ^†^
Soltannezhad et al. (2013) [53]	Y	UC	Y	Y	Y	N	Y	Y	Y	Y	Y	Y	UC	10/13 ^†^
Song et al. (2022) [54]	Y	Y	Y	UC	UC	UC	Y	UC	Y	Y	Y	Y	UC	8/13 ^†^
Wesołowska-Górniak et al. (2022) [55]	Y	Y	Y	UC	UC	Y	UC	UC	UC	Y	Y	Y	UC	7/13 ^†^
**Quasi-experimental studies**														
Chambord et al. (2021) [35]	Y	Y	Y	Y	Y	UC	Y	Y	Y	-	-	-	-	8/9 ^‡^
Enrico et al. (2018) [37]	Y	N/A	N/A	N	Y	N/A	Y	Y	Y	-	-	-	-	5/9 ^‡^
Hoseinian et al. (2023) [38]	Y	Y	Y	Y	Y	Y	Y	Y	Y	-	-	-	-	9/9 ^‡^
Jabeen et al. (2021) [40]	Y	N/A	N/A	N	Y	N/A	Y	Y	Y	-	-	-	-	5/9 ^‡^
Jeong et al. (2021) [41]	Y	N/A	Y	N	Y	N/A	Y	Y	Y	-	-	-	-	6/9 ^‡^
Kim et al. (2017) [42]	Y	Y	Y	Y	Y	Y	Y	Y	Y	-	-	-	-	9/9 ^‡^
O’Brien et al. (2024) [47]	Y	Y	Y	N	Y	Y	Y	Y	Y	-	-	-	-	8/9 ^‡^
Sim et al. (2012) [52]	Y	N/A	Y	N	Y	Y	Y	Y	Y	-	-	-	-	7/9 ^‡^
Thangto et al. (2022) [21]	Y	Y	Y	N	N	UC	Y	Y	Y	-	-	-	-	6/9 ^‡^
Xie et al. (2023) [56]	Y	Y	Y	Y	N	Y	Y	Y	Y	-	-	-	-	8/9 ^‡^
**Mixed-method studies**														
Been-Dahmen et al. (2019) [34]	Y	UC	Y	Y	Y	UC	Y	Y	Y	-	-	-	-	7/9 ^‡^

N = no; N/A = not applicable; RCT = randomized controlled trial; UC = unclear; Y = yes; ^†^ JBI Critical Appraisal Checklist for Randomized Controlled Trials (min. 0 points; max. 13 points) includes the following items; Q1 = Was true randomization used for assignment of participants to treatment groups?; Q2 = Was allocation to treatment groups concealed?; Q3 = Were treatment groups similar at the baseline?; Q4 = Were participants blind to treatment assignment?; Q5 = Were those delivering treatment blind to treatment assignment?; Q6 = Were outcomes assessors blind to treatment assignment?; Q7 = Were treatment groups treated identically other than the intervention of interest?; Q8 = Was follow up complete and if not, were differences between groups in terms of their follow up adequately described and analyzed?; Q9 = Were participants analyzed in the groups to which they were randomized?; Q10 = Were outcomes measured in the same way for treatment groups?; Q11 = Were outcomes measured in a reliable way?; Q12 = Was appropriate statistical analysis used?; Q13 = Was the trial design appropriate for the topic, and any deviations from the standard RCT design (individual randomization, parallel groups) accounted for in the conduct and analysis of the trial? ^‡^ JBI Critical Appraisal Checklist for Quasi-Experimental Studies (min. 0 points; max. 9 points) includes the following items; Q1 = Is it clear in the study what is the ‘cause’ and what is the ‘effect’ (i.e., there is no confusion about which variable comes first)?; Q2 = Were the participants included in any comparisons similar?; Q3 = Were the participants included in any comparisons receiving similar treatment/care, other than the exposure or intervention of interest?; Q4 = Was there a control group?; Q5 = Were there multiple measurements of the outcome both pre and post the intervention/exposure?; Q6 = Was follow up complete and if not, were differences between groups in terms of their follow-up adequately described and analyzed?; Q7 = Were the outcomes of participants included in any comparisons measured in the same way?; Q8 = Were outcomes measured in a reliable way?; Q9 = Was appropriate statistical analysis used?

**Table 3:** Self-management intervention.

Intervention Type	Study First Author (Year)	Providers	Target	Sessions (Duration)	Intervention Contents
Education	Aghakhani et al. (2021) [32]	Nurse	KT patients	3 sessions of 30 to 45 min (3 weeks)	IG received face-to-face education programs using an educational booklet. The content of the program included the type of disease, medication, diet, and physical and self-care activities.
	Côté et al. (2018) [36]	Nurse/web	KT patients	3 sessions, each 20 to 30 min (3 months)	Transplant-TAVIE, a web-based tailored nursing intervention that empowers kidney transplant recipients to manage their immunosuppressive drug treatment. The sessions aimed to help users incorporate the therapeutic regimen into their daily routine, cope with medication side effects, handle situations or circumstances that could interfere with medication intake, interact with healthcare professionals, and mobilize social support. The learning objectives included strengthening various capacities such as self-motivation and self-monitoring (session 1), problem-solving and emotional control (session 2), and social interaction (session 3).
	Hoseinian et al. (2023) [38]	Nurse	Patients undergoing KT	8 sessions (2 months)	- Education in the intervention group was based on the model of health promotion strategies. The intervention method was such that each individual patient was educated using the methods of lecture, discussion, and question and answer. - The patients of the intervention group were educated through health promotion strategies and considering three levels of prevention in the areas of stress reduction, adaptation, decision making, enjoying life, activity, rest, nutrition, and medication.
	Hu et al. (2022) [20]	Nurse	KT patients (≥3 months)	3 sessions (3 months)	One session was conducted each month. (1) Drug-taking manual: the benefits of transplantation, the necessity of taking immunosuppressive drugs, the consequences of taking immunosuppressive drugs, the methods of administration, and matters associated with various immunosuppressive drugs requiring attention regarding (e.g., medication schedule). (2) Methods of blood concentration monitoring and matters requiring attention to keep the blood concentration stable, the consequences of rejection (a small amount), the occurrence and treatment of infection (overdose) after taking immunosuppressive drugs, and behavior feedback results of the first month. (3) Prevention and treatment of complications, including matters requiring attention such as self-protection and modification of lifestyle, as well as the introduction of self-monitoring indicators.
	Kim et al. (2017) [42]	Nurse	KT patients	6 sessions, 60 min per session (6 weeks)	The topics discussed during educational sessions were as follows: (1) program introduction, (2) drug administration, (3) symptoms of rejection and complications, (4) nutritional management, (5) exercising, and (6) management of activities of daily living. The program was delivered through education, listening, conversation, linguistic support, raising issues, and seeking solutions.
	Kuwaiti et al. (2018) [43]	Nurse, psychologist, nutritionist	KT patients (≥3 months)	2.5 hr sessions; 15 hr comprehensive program (6 weeks)	The content of the program included the following: (1) Techniques for addressing problems such as frustration, fatigue, and isolation. (2) Good exercise for maintaining and boosting strength. (3) The efficient use of medications. (4) An effective relationship with family, friends, and healthcare specialists. (5) Nutrition. (6) Process of evaluating new treatments, with emphasis on operational planning, problem-solving, and decision-making.
	Mollazadeh et al. (2018) [45]	Nurse	KT patients (3–12 months)	5 sessions, 60 min per session (3 months)	The contents include self-monitoring and self-care behavior in daily living, early detection and coping with abnormalities after KT, stress management, and management of non-categorized cases. The intervention also consists of an assessment of the patient’s need for self-management education and intensive training that verifies understanding by verbalizing what has been explained.
	Othman et al. (2024) [48]	Nurse, public health worker, pharmacist	KT patients (≥6 months)	6 session, first session: 60 min, other sessions: 30 min (3 months)	The education content was repeated entirely or partly by the researcher according to each patient’s needs. Mixed education techniques, such as description, question, and answer, were used as an education method, and feedback was stimulated to enable patients to understand their self-care management independently.
	Robinson et al. (2015) [50]	Audio support education delivered by tablet	KT patients (2–24 months)	1 session, 23–42 min (waiting time to consult with a nephrologist)	The topics included were as follows: (1) importance of sun protection, (2) skin cancer, (3) risk of developing skin cancer, (4) ways people get sun exposure, (5) choices of sun protection, (6) frequently asked questions about sunscreen, (7) protective clothing, and (8) personalized sun-protection recommendations.
	Soltannezhad et al. (2013) [53]	Physician	Patients undergoing KT	4 sessions, 30 min per session (2 months)	-Educational sessions included topics on stress management, coping strategies, nutrition, exercise, medication adherence, and self-care after surgery, and common complications. -Educational booklet and researcher’s telephone numbers were provided in cases where participants have probable questions. -An educational pamphlet was provided to the family.
	Song et al. (2022) [54]	Doctor, nurses, dieticians	Patients undergoing KT	FTS nursing and continuous nursing (6 months)	-The FTS nursing activities were as follows; (1) personalized care plan, (2) preoperative education, (3) intraoperative preparation, (4) postoperative observation, and (5) guidance at discharge. -The continuous nursing activities were as follows; (1) patient follow-up after discharge, (2) gathering of patient’s files, (3) provision of self-management education, (4) personalized adjustment of nursing plan, and (5) provision of psychological care.
	Thangto et al. (2022) [21]	Nurse, dietitian, pharmacist	Patients undergoing KT	3 sessions, 1 h per session (hospitalization)	-The education program consisted of three major sessions covering the three key area of knowledge self-management and care (taught by clinical nurses), nutrition and diet (taught by dietitians), and immunosuppressive drugs (taught by pharmacists).
Education, exercise intervention	Schmid-Mohler et al. (2019) [51]	Nurse	KT patients (≤6 weeks)	8 or 9 sessions, session 1: 45–60 min sessions 2–3: 45–60 min sessions 4–9: 15–30 min (12 months)	-Educational intervention included as follows: medication self-management, emotional and psycho-social concerns, weight management, physical activity, and recommendations regarding diet and activity. -Behavior intervention was focused on maintenance/achievement of a normal body weight and the integration of physical activity into the daily routine.
Exercise intervention	Enrico et al. (2018) [37]	N/A	KT patients (≥12 months)	3 times a week for 60 min per session (12 months)	The program consisted of aerobic and strength training exercises. An initial goal included achieving 30 min of exercise at least 3 times a week, progressing to 150 min a week of moderate physical exercise by the sixth month, and continuing for the duration of the study. The exercise sessions included strength exercises to enhance the function of eight muscle groups that have been affected in our diseased populations and 15 min of stationary cycling. Diet: The patients were advised to follow a traditional Mediterranean nutritional diet that included consumption of at least two daily portions of fruit and three servings of vegetables, as well as three servings of fish per week and two servings of cereal per week.
	O’Brien et al. (2020) [46]	Nurse /Mobile phone	KT patients (≥3 months)	Daily usage of SystemCHANGE and activity tracker (6 months)	The personal system-based solutions support and enhance physical activity combined with visual feedback from a mobile activity tracker. SystemCHANGE + activity tracker intervention focuses on guiding individuals to incorporate the desired behavior change as part of their daily or weekly routines.
	Wesołowska-Górniak et al. (2022) [55]	Nurse, physician	KT or LT patients (1–5 years)	N/A (3 months)	The patients were required every day to monitor their daily physical activity using a pedometer and to complete a diary of their daily number of steps.
Counselling	Barchfeld et al. (2023) [33]	Physician, clinical psychologist	KT patients (≥3 months)	12 sessions, each 50 min (6 months)	- Sessions were offered face-to-face or telemedically (via video conferencing or telephone). The intervention included cognitive–behavioral as well as psychoeducational elements. - The content of the intervention included the following: Nutritional and exercise counselling, overweight and obesity, reasons for and against losing weight, eating cues, progress report, resources and behavioral change, mindfulness, vicious circle, stress management, repetition, relapse prevention.
Education, counselling	Sim et al. (2012) [52]	Nurse	KT patients (≥ 1 months)	2 sessions, each 50–60 min (2 weeks)	The topics discussed during educational sessions were as follows: (1) medication and laboratory tests as well as complications and preventive measures, and (2) dietary management, general health management, and exercise.
	Jeong et al. (2021) [41]	Nurse	Patients who transferred to the ward after KT	2 sessions, 30 min per session (2 weeks)	Video education included the following content areas: (1) medication, (2) nutrition, (3) exercise, (4) rejection, and (5) complications. Personalized counselling included the following content areas: (1) daily activity, (2) social support, (3) emotional support, (4) self-care motivation, and (5) self-care planning.
Mobile phone-based medication monitoring	McGillicuddy et al. (2013) [44]	Mobile phone	KT patients (≥3 months)	N/A (3 months)	IG received customizable reminder signals (light, chime), phone calls, or text messages at the prescribed dosing day and time. They were contacted by text, email, or phone when alerts indicated medication non-adherence. A weekly summary report was submitted via email, and a summary of each participant’s adherence to medication dosing was provided by a physician.
Mobile app	Xie at al. (2023) [56]	Mobile app, nurse	KT patients	N/A	The intervention group used a mobile application to implement self-management behavioral interventions were as follows: (1) Information support: 11 categories of health knowledge related to each stage of Renal Transplantation, (2) Skills guidance: Provide intelligent self-monitoring forms to facilitate patients in recording important data such as daily blood pressure, body weight, and intake and output volume, and provide automatic conversion of water content of each food, intake and output volume statistics, medicine and food, (3) Communication: self-monitoring data and medical side sharing, convenient for nurses and patients at any time to exchange disease changes, seek help, and share disease-related monitoring data.
	O’Brien et al. (2023) [47]	Mobile app	KT patients	N/A (3 months)	The intervention began with the development of a “Plan” (individual goals for dietary intake and minutes of physical activity), and the participant identified possible ways (personalized solutions based on their everyday routines) to achieve daily goals. For the “Do” component, participants incorporated their personalized solutions into existing routines. The “Study” component enabled the participants to evaluate their dietary and physical activity goal progress with visual feedback (graphs) from the Lose-It© app. The “Act” phase enabled the participants to evaluate the personalized-system solution and determine the achievement of the dietary and physical activity goals. During the session weeks 1–12, the participant completed four steps of the Plan-Do-Study-Act Model with the RA via Zoom.
Mobile app, nursing coaching	Pollock et al. (2023) [49]	Mobile app, nurse	KT patients (≥ 1 months)	N/A (3 months)	The MyKidneyCoach intervention comprised (1) a self-management app that provided educational materials and monitoring of post-transplant care using a smartphone and (2) personalized, text-based, and telephonic coaching from a trained clinical nurse triggered through the app to promote patient activation and self-management after KT.
Enhancing motivation intervention	Been-Dahmen et al. (2019) [34]	Nurse/web	KT patients (1–8 months)	4 sessions (dependent on individual)	In the first session, self-management challenges were assessed using a self-management web-based program, specifically designed for this purpose. Progression toward goal attainment and outcome expectations were discussed in the second and third sessions. Goal progress, relapse prevention, and generalization of learned skills to other challenges were discussed during the fourth session.
Enhancing empowerment intervention	Hsiao et al. (2016) [39]	Nurse	KT patients (≤20 years)	Six small-group sessions, each lasting for 120 min (12 weeks)	Topics included setting goals, solving problems, coping with renal transplant, coping with daily stresses, seeking social support, and staying motivated. The sessions consisted of introductions that highlighted the topic, group discussions, and patient identification of problem areas for self-care behaviors after renal transplantation. Furthermore, the emotions associated with these problems were explored, and a set of goals and strategies to overcome these problems was developed. Active learning (sharing experiences with others and choosing personal solutions) was encouraged.
Self-management support intervention	Jabeen et al. (2021) [40]	Nurse	KT patients	3 sessions, 45–60 min per session (4 months)	Three sessions; (1) nurses training, (2) applying the program to patients, and (3) assessment. The content of the program included management of the expected health problems; performance of routine activities; management of emotional changes, such as stress, anxiety, fear, and depression; dietary modifications; management of sleep pattern; and maintenance of effective communication with family and colleagues.
Behavioral educational interview intervention	Chambord et al. (2021) [35]	Pharmacist	KT patients (4–12 months)	30 min, 11 scenarios (4 months)	It was performed at visit 1 in the IG patients, consisted of a behavioral and educational interview. Using the Barrows cards, the patient was provided with a ‘situation’ that represents a problem they might encounter concerning immunosuppressive treatment or pathology. The patient selected one of the three ‘behavior’ cards according to the reaction they would have adopted if placed in this situation. The consequences of the choice were then discussed with the pharmacist leading the interview.

FTS = fast-track surgery; IG = intervention group; KT = kidney transplantation; LT = liver transplantation; min = minutes; N/A = Not applicable.

**Table 4:** Categorization and summary of outcomes.

Category	Positive Outcomes	Null Outcomes	Mixed Outcomes ^†^
**Cognitive Outcomes**	Treatment knowledge (*p* < 0.001) [35], self-management knowledge (*p* < 0.001) [21,48,52], knowledge of skin cancer and sun protection (*p* = 0.04) [50], perception of care (*p* < 0.001) [51], quality of life (*p* < 0.05) [33,40,43,54,56], (*p* < 0.001) [20,32]	Self-perceived general health status [36], quality of life [34]	Quality of life
**Behavioral Outcomes**	Self-care behavior (*p* = 0.009) [39], (*p* < 0.05) [56], (*p* < 0.001) [40,45], diabetes self-care (*p* < 0.05) [48], medication adherence (*p* < 0.05) [20,54], (*p* < 0.001) [44], self-care ability (*p* < 0.05) [20,54], self-care self-efficacy (*p* < 0.001) [38,41,48], (*p* < 0.05) [56], daily steps (*p* = 0.03) [46], self-efficacy to exercise (*p* = 0.003) [47], sun protection behavior (*p* = 0.01) [50], and treatment compliance (*p* < 0.001) [42]	Self-care behavior [34], medication adherence [35,36], measurement of drug exposure [35], medication intake-related skills [36], self-care self-efficacy [34,36,53], physical activity [51,55], average daily number of steps within 7 days [55], fruits/vegetable intake, fiber intake, sodium intake [47]	Self-care behavior, medication adherence, self-care self-efficacy, and daily steps
**Affective Outcomes**	Feelings after kidney transplantation, adherence to immunosuppressive medications (*p* = 0.03) [34], perceived level of empowerment (*p* = 0.023) [32], satisfaction level (*p* < 0.05) [20], self-perceived burden (*p* < 0.05) [20], anxiety/depression (*p* < 0.05) [20], stress (*p* = 0.04) [47], autonomy support (*p* = 0.038) [41], competence (*p* < 0.001) [41], attitudes (*p* = 0.02) [50], and uncertainty (*p* < 0.001) [42]	Feelings after KT, worry about the transplantation/guilt toward the donor/disclosure about the transplantation/responsibility toward others [34], quality of nurse-led care [34], social support [34], and level of patients’ comfort [54], anxiety/depression [33]	Anxiety/depression
**Health Outcomes**	Systolic blood pressure (*p* = 0.009) [44], diastolic blood pressure (*p* = 0.00006) [44], HR (*p* = 0.002) [46], WC (*p* < 0.001) [46,48], and BMI (*p* = 0.01) [46], (*p* < 0.05) [33], weight (*p* < 0.05) [33], (*p* = 0.02) [47], HbA1c (*p* < 0.05) [48], proteinuria (*p* = 0.016) [48]	Blood pressure [46,47], weight [46], BMI [51,55], body composition LTM [51], 6MWT [46], medication side effects [36], myocardial function [37], renal function parameters [33]	Blood pressure, BMI, and renal function

6MWT = 6 min walk test, BMI = body mass index, HR = heart rate, KT = kidney transplantation, LTM = lean tissue mass, WC = waist circumference; ^†^ Mixed outcomes refer to variables presented in both positive and negative outcomes.

## Data Availability

The dataset used and/or analyzed during this study can be provided by the corresponding author upon reasonable request.

## Supplementary Materials

The following supporting information can be downloaded at: https://www.mdpi.com/article/10.3390/healthcare13151918/s1, Table S1: PRISMA 2020 Checklist; Table S2: PRISMA 2020 for Abstracts Checklist; Supplementary Materials S3: International prospective register of systematic reviews; Supplementary Materials S4: Example search strategy. Reference [67] is cited in the Supplementary Materials.
