# Supplementary material for: Self-Management Interventions for Kidney Transplant Recipients: A Systematic Review
Source: Healthcare (Basel). 2025 Aug 5;13(15):1918. doi: 10.3390/healthcare13151918 (PMC12345960; doi:10.3390/healthcare13151918)
Supplement: Supplementary file 1 [file healthcare-13-01918-s001.zip › Supplementary Materials S4.pdf]

S4. Example search strategy

1) MEDLINE via Pubmed

1. ("kidney transplant*"[Text Word]) OR ("renal transplant*"[Text Word])) OR ("kidney replace*"[Text Word]) OR ("kidney graft"[Text Word]) OR ("renal graft"[Text Word]) OR (kidney transplantation[MeSH Terms])
2. ("self management"[Text Word]) OR ("self care"[Text Word]) OR ("self monitoring"[Text Word])) OR ("self administration"[Text Word])) OR ("self guided"[Text Word])) OR ("self help"[Text Word])) OR ("self directed"[Text Word]) OR ("self regulate"[Text Word]) OR (self management [MeSH Terms])
3. #1 AND #2

2) Embase

('kidney transplantation'/exp OR 'kidney graft'/exp OR 'kidney allograft'/exp) AND ('self care'/exp OR 'self help'/exp OR 'self monitoring'/exp OR 'drug self administration'/exp OR 'self-directed learning'/exp)

3) CINAHL

("kidney transplant*" OR "renal transplant*" OR "kidney graft" OR "renal graft") AND ("self management" OR "self care" OR "self monitoring" OR "self administration" OR "self guided" OR "self help" OR "self directed" OR "self regulate")

4) Cochrane

#1 ("kidney transplant*" OR "renal transplant*" OR "kidney replace*" OR "kidney graft" OR "renal graft") (Word variations have been searched)

#2 MeSH descriptor: [Kidney Transplantation] explode all trees

#3 ("self management" OR "self care" OR "self monitoring" OR "self administration" OR "self guided" OR "self help" OR "self directed" OR "self regulate") (Word variations have been searched)

#4 MeSH descriptor: [Self-Management] explode all trees

#5 #1 OR #2

#6 #3 OR #4

#7 #5 AND #6
